# Corrigendum: Longitudinal associations between microRNAs and weight in the diabetes prevention program

**DOI:** 10.3389/fendo.2024.1511917

**Published:** 2024-12-04

**Authors:** Elena Flowers, Benjamin Stroebel, Xingyue Gong, Kimberly A. Lewis, Bradley E. Aouizerat, Meghana Gadgil, Alka M. Kanaya, Li Zhang

**Affiliations:** ^1^ Department of Physiological Nursing, University of California, San Francisco, San Francisco, CA, United States; ^2^ Institute for Human Genetics, University of California, San Francisco, San Francisco, CA, United States; ^3^ Bluestone Center for Clinical Research, New York University, New York, NY, United States; ^4^ Department of Oral and Maxillofacial Surgery, New York University, New York, NY, United States; ^5^ Division of General Internal Medicine, Department of Medicine, University of California, San Francisco, San Francisco, CA, United States; ^6^ Department of Epidemiology and Biostatistics, University of California, San Francisco, San Francisco, CA, United States; ^7^ Division of Hematology and Oncology, Department of Medicine, University of California, San Francisco, San Francisco, CA, United States

**Keywords:** microRNA, diabetes, weight loss, biomarker, prediabetes

In the published article, there was an error in the author list, and author Xingyue Gong was listed in the wrong order. The corrected author list appears below.

“Elena Flowers^1,2^, Benjamin Stroebel^1^, Xingyue Gong^1^, Kimberly A. Lewis^1^, Bradley E. Aouizerat^3,4^, Meghana Gadgil^5^, Alka M. Kanaya^5,6^, Li Zhang^6,7^”

The authors apologize for this error and state that this does not change the scientific conclusions of the article in any way. The original article has been updated.

