# Supplementary figures and images for: Longitudinal associations between microRNAs and weight in the diabetes prevention program
Source: Front Endocrinol (Lausanne). 2024 Sep 18;15:1419812. doi: 10.3389/fendo.2024.1419812 (PMC11445047; doi:10.3389/fendo.2024.1419812)

**Supplementary Figure 1.** Upset Plot for Predicted KEGG Pathways by Combination of MicroRNAs


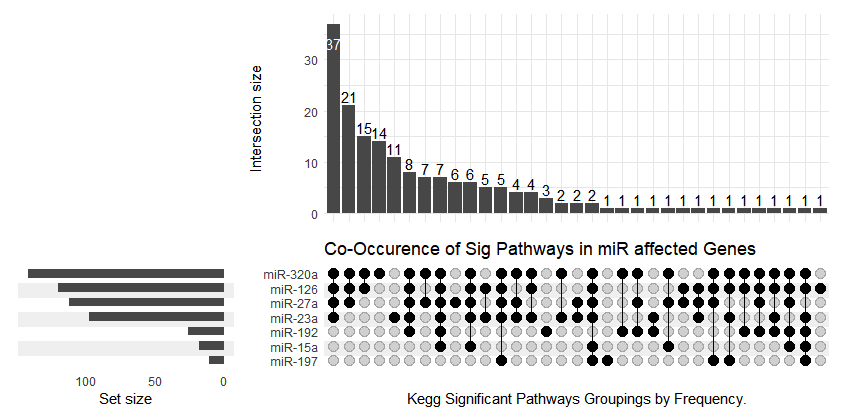

Supplement: Supplementary Figure 1 — This upset plot depicts the overlap in the pathways that were enriched for mRNA targets of the 7 microRNAs that were significantly associated with weight over 2 years. Intersection size is the number of pathways targeted by the combination of microRNAs indicated by black dots below. Numbers above dark grey bars represent the intersection size value. Set size is the total number of pathways targeted by each individual microRNA. [file DataSheet1.docx]
